# Early Postnatal Genistein Administration Affects Mice Metabolism and Reproduction in a Sexually Dimorphic Way

**DOI:** 10.3390/metabo11070449

**Published:** 2021-07-10

**Authors:** Marilena Marraudino, Giovanna Ponti, Chantal Moussu, Alice Farinetti, Elisabetta Macchi, Paolo Accornero, Stefano Gotti, Paloma Collado, Matthieu Keller, Giancarlo Panzica

**Affiliations:** 1 Neuroscience Institute Cavalieri Ottolenghi (NICO), Regione Gonzole 10, Orbassano, 10043 Torino, Italy; marilena.marraudino@unito.it (M.M.); gponti2@gmail.com (G.P.); alice.farinetti@unito.it (A.F.); stefano.gotti@unito.it (S.G.); 2 UMR Physiologie de la Reproduction et des Comportements, Institut National de Recherche pour l’agriculture, l’Alimentation et l’Environnement (INRAE), Centre National de la Recherche Scientifique (CNRS), Institut Français du Cheval et de l’Equitation (IFCE), Université de Tours, 37380 Nouzilly, France; chantal.porte@inrae.fr (C.M.); matthieu.keller@inrae.fr (M.K.); 3 Laboratory of Neuroendocrinology, Department of Neuroscience Rita Levi Montalcini, University of Torino, Via Cherasco 15, 10125 Torino, Italy; 4 Department of Veterinary Sciences, University of Torino, Largo Braccini 2, Grugliasco, 10095 Torino, Italy; elisabetta.macchi@unito.it (E.M.); paolo.accornero@unito.it (P.A.); 5 Department of Psychobiology, Universidad Nacional de Educación a Distancia (UNED), C/Juan del Rosal 10, 28040 Madrid, Spain; pcollado@psi.uned.es

**Keywords:** phytoestrogens, endocrine disruptor, dimorphism, obesity, kisspeptin, POMC, orexin

## Abstract

The phytoestrogen genistein (GEN) may interfere with permanent morphological changes in the brain circuits sensitive to estrogen. Due to the frequent use of soy milk in the neonatal diet, we aimed to study the effects of early GEN exposure on some physiological and reproductive parameters. Mice of both sexes from PND1 to PND8 were treated with GEN (50 mg/kg body weight, comparable to the exposure level in babies fed with soy-based formulas). When adult, we observed, in GEN-treated females, an advanced pubertal onset and an altered estrous cycle, and, in males, a decrease of testicle weight and fecal testosterone concentration. Furthermore, we observed an increase in body weight and altered plasma concentrations of metabolic hormones (leptin, ghrelin, triiodothyronine) limited to adult females. Exposure to GEN significantly altered kisspeptin and POMC immunoreactivity only in females and orexin immunoreactivity in both sexes. In conclusion, early postnatal exposure of mice to GEN determines long-term sex-specific organizational effects. It impairs the reproductive system and has an obesogenic effect only in females, which is probably due to the alterations of neuroendocrine circuits controlling metabolism; thus GEN, should be classified as a metabolism disrupting chemical.

## 1. Introduction

Genistein (GEN; 4′,5,7-trihydroxyisoflavone) is an isoflavonoid compound. Its chemical structure shares features with 17β-estradiol, enabling it to bind to estrogen receptors. GEN is produced by many plants, is highly present in Leguminosae species, and, due to its estrogenic activity, is considered a phytoestrogen [1]. The main sources of GEN, in our diet, are soybeans and soy-based foods. Most foods contain a small quantity of isoflavones, but when consumed regularly and from various sources, they can reach a cumulative dose that can contribute to long-term effects [2]. In adults, phytoestrogens, including GEN, have been generally associated with beneficial effects, i.e., obesity and diabetes [3], menopause [4], cancer [5], and hypertension associated to metabolic syndrome [6]; however, many studies suggest that phytoestrogens are harmful to human health [7,8]. Above all, babies may be subjected to higher levels of phytoestrogens (6–9 mg/kg/day) than typical adult exposures (approximately 1 mg/kg/day) [9]. In fact, in addition to soy-based infant formulas also many soy-based foods are specifically prepared for babies [9,10]. In rodents, sex-specific alterations were reported in several neuronal populations, including hypothalamic and amygdaloid circuits containing many gonadal hormone-sensitive neurons [11,12,13,14,15,16].

The neuroendocrine control of food intake and energy expenditure is based on many circuits including both orexinergic and anorexinergic elements that are targets for a series of chemical signals coming from the periphery (for a review see [17]). The main centers of this control are the arcuate nucleus (ARC), the dorsomedial hypothalamic nucleus (DMH), the paraventricular hypothalamic nucleus (PVN), and the lateral hypothalamus (LH). The ARC contains orexinergic elements (NPY/AgRP neurons) and anorexinergic ones (the pro-opiomelanocortin/cocaine- and amphetamine-regulated transcript neurons (POMC/CART)) while the LH encompasses orexin-A/hypocretin-1 (OX) neurons. Unlike NPY/AgRP neurons (stimulating food intake), OX stimulates both feeding and energy expenditure, in response to physiological variation in glucose blood levels between meals [18]. Among the peripheral signals, leptin, a satiety hormone produced by adipocytes, stimulates POMC neurons in the ARC [19] and depresses the OX system. In the ARC, OX fibers contact POMC cells decreasing their synthesis and promoting hyperphagia and weight gain [20]. Interestingly, POMC and OX fibers directly project to the PVN, the most important center of metabolic control. The PVN expresses the receptor for OX [21] and the two peptides released from POMC neurons: melanocortin and α-melanocyte-stimulating hormone (α-MSH) [22]. Moreover, the PVN modulates metabolism through its action on the hypothalamus-pituitary-adrenal and on the hypothalamus-pituitary-thyroids axis [23]. The medial part of the PVN, where corticotropin-releasing hormone (CRH) and thyrotropin-releasing hormone (TRH) neurons are located, is strongly innervated by kisspeptin (kiss) fibers [24]. The kiss system is formed by a neuronal population more numerous in females than in males [25], and, in rodents, kiss cells are clustered in the ARC and the rostral periventricular area of the third ventricle (RP3V). This peptide was, at first, identified as one of the key controllers of GnRH neurons (for a review see [26]), but today, kiss neurons seem to have a major nodal role in integrating the different signals transmitting metabolic information, from both the periphery and central nervous system, onto reproductive centers [27]. The connection between energy balance, puberty, and reproduction is evident, particularly in females, in conditions of anorexia (energy insufficiency) or obesity (excess of energy) [27]. Kiss neurons at the time of puberty are directly modulated by leptin [28]. Hypothalamic colocalization of kiss and leptin receptors is controversial and limited only to the ARC group; in particular, the estimated degree of colocalization varies between 40% [29], 15% [30], and 6–8% [31] of the kiss neurons in the mouse ARC. In addition, the signaling system becomes fully mature after the completion of puberty [29,30]. Moreover, leptin deficiency reduces kiss mRNA expression in the ARC [29,32] and the number of positive neurons in the RP3V [32]. Finally, kiss has strong effects on both POMC and NPY systems in the ARC: kiss has an excitatory effect on POMC neurons via the kiss receptor [33], which is expressed in about 63% of POMC-immunoreactive neurons in female rats [34] and inhibits NPY cells via the GABA system [33]. However, in vitro, kiss induces an increase of NPY and a decrease of BDNF expression [35]. In addition to peripheral stimuli such as leptin and other hormones produced by the gastrointestinal system (i.e., Ghrelin and others), estrogen also plays an important role in controlling food intake and metabolism [36].

Hypothalamic circuits involved in the regulation of food intake and energy metabolism are therefore potential targets for xenoestrogens, including phytoestrogens and GEN [37]. Much evidence suggests that they could have an obesogenic effect [36] or might counteract aspects of the metabolic syndrome [38]. Treatment with GEN affects body weight in rats, but the effect depends on sex, age, and hormonal status [38,39]. In the young, in ovariectomized [40,41] and intact adult female mice [42] GEN shows an anti-obesogenic effect, but this appears to depend on the dose: adipogenesis is inhibited at low doses of GEN [43]. However, in males, the low concentration has an obesogenic effect [44]. In rats, the perinatal treatment of GEN has different effects: obesogenic in females [45] and anti-obesogenic in males [46].

The ability of GEN to bind the estrogen receptors (ERs) [12] makes the hypothalamic circuits highly sensitive to this molecule. A large body of literature is present on the effect of GEN on the kiss system with in vitro [47] and in vivo studies (for a review see [48]). No significant alterations of the kiss system were found in adult male rats postnatally exposed to GEN [49], while in females this system was mostly affected with a reduction of fiber density [11,50]. Yet, little is known about the GEN effects on the circuits that control food intake and energetic metabolism. A single study [51] shows that high phytoestrogens (daidzein and genistein) concentrations decrease, in male mice hypothalamus, AgRP and increase MCH, orexin A, and TRH mRNA levels in postnatal life, but it does not influence NPY, POMC, and CART expression. However, in rats, the postnatal administration of two doses of GEN (10 or 50 µg/g body weight) from PND6 to PND13 decreased the number of POMC-ir cells in the ARC only in females, while the OX system did not appear to be affected by the treatment in either sex [52].

Our study aimed to enclose in a single experiment the potential for sexually dimorphic effects of early postnatal administration of GEN in mice (at a dose comparable to that of babies fed with soy-based formulas) on: (I) female reproductive peripheral parameters (puberty onset, estrus cycle, mammary gland development); (II) male reproductive peripheral parameters (testosterone level, testicle size); (III) control of food intake and metabolic regulation through hormonal (leptin, triiodothyronine, ghrelin) and physiological (body weight, food consumption, and feed efficiency) parameters; (IV) hypothalamic circuits involved in both reproductive and metabolic control (POMC, orexin, kisspeptin systems).

## 2. Results

### 2.1. Female Reproductive Parameters

#### 2.1.1. Vaginal Opening and Estrous Cycle

**Figure 1:**
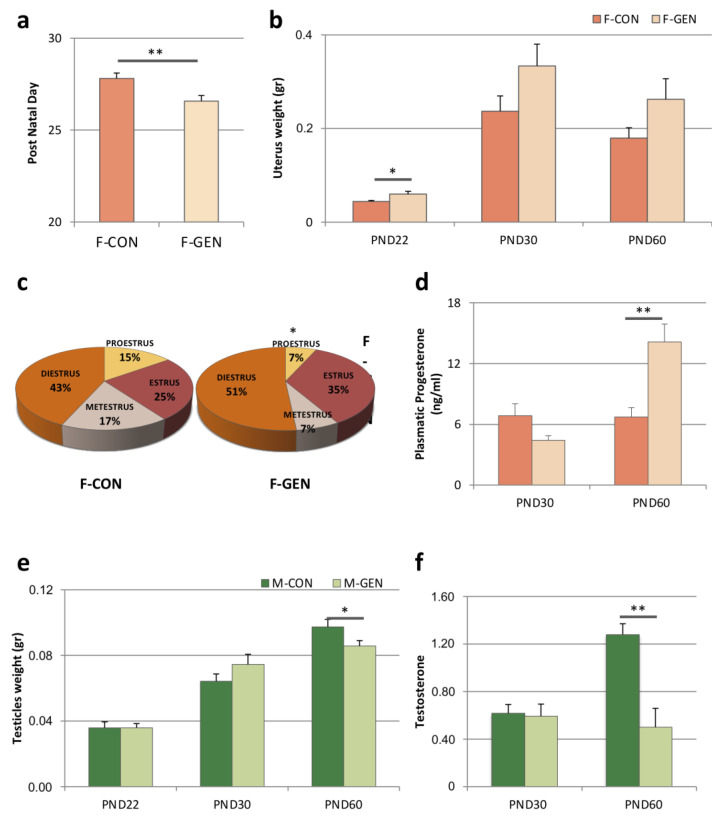
Parameters related to reproduction. (**a**) Histograms representing the evaluation of the day of vaginal opening (VO) in control (F-CON) and treated (F-GEN) female CD1 mice (mean ± SEM). (**b**) Histograms representing the variations of the uterus weight (expressed in gram; mean ± SEM) measured during development at postnatal day (PND) 22, PND30, and PND60 of control (F-CON) and treated (F-GEN) females. (**c**) Pie charts illustrating the time spent (expressed as percentage) in each phase of estrus cycle (estrus, metestrus, diestrus, and proestrus) in control (F-CON) and treated (F-GEN) females. (**d**) Histograms representing circulating variations of plasma concentration of progesterone (expressed in ng/mL, mean ± SEM) in control (F-CON) and treated (F-GEN) females, during development at PND30 and PND60. (**e**) Histogram representing the increase of testicle weight (expressed in gram; mean ± SEM) measured during development at postnatal day (PND) 22, PND30, and PND60 of control (M-CON) and treated (M-GEN) males. (**f**) Histogram representing variations of fecal testosterone levels (expressed in ng/mL) in control (M-CON) and treated (F-GEN) males, during development at PND30 and PND60. * *p* < 0.05; ** *p* ≤ 0.01. VO, uterus and testicles weight, and time spent in each phase of estrus cycle were compared using Student’s *t*-test, while the progesterone was analyzed using Tukey’s test.

Vaginal opening (VO) is one of the parameters employed to evaluate the potential effects of endocrine disrupting chemicals on puberty onset in rodents [53]. GEN treatment stimulated an anticipation of the VO (F-CON = 27.8 ± 0.29; F-GEN = 26.5 ± 0.30, *p* = 0.005) (Figure 1a).

GEN treatment altered the estrous cycle: F-GEN spent more time in estrus and diestrus phases compared to F-CON (Estrus = 30% and Diestrus = 43% vs. 35% and 51% respectively in F-GEN) with a significant reduction of proestrus phase (*t*-Test, *p* = 0.03; F-CON = 16.67 vs. F-GEN = 6.67) (Figure 1c).

#### 2.1.2. Uterus Weight

The uterus weight of F-GEN was higher than F-CON starting from PND22, however, the difference was significant only at PND22 (Student’s *t*-test *p* = 0.035), but not after puberty (PND30, *p* = 0.120 and PND60, *p* = 0.123) (Figure 1b). Two-way ANOVA for age and treatment as independent variables confirmed this result (respectively F = 30.489, *p* = 0.001; F = 6.797, *p* = 0.014), but no difference was present in their interaction (F = 0.951, *p* = 0.398).

#### 2.1.3. Progesterone Level

Progesterone serum levels in control females did not change significantly in young (PND30 6.84 ± 1.19) and adult (PND60 6.73 ± 0.90; *p* = 0.565) animals. GEN treatment did not affect progesterone levels in young animals (PND30 F-CON vs. F-GEN; *p* = 0.41), while it significantly increased it in adults (PND60 F-CON vs. F-GEN; *p* = 0.007) (Figure 1d).

#### 2.1.4. Mammary Gland Analysis

**Figure 2:**
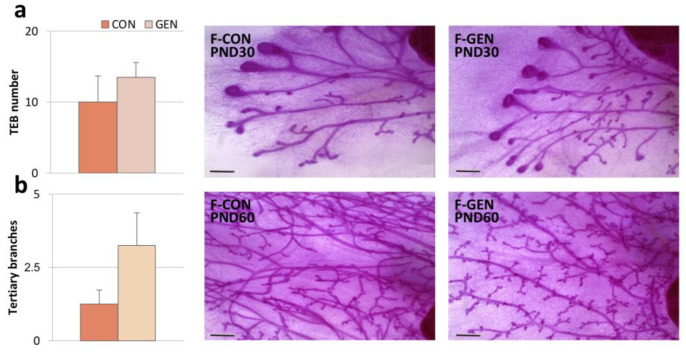
Mammary gland. (**a**) Histograms (**left**) and photomicrograph (**right**) showing the number of terminal end buds (TEBs) of mammary glands in control (CON) and treated (GEN) female CD1 mice at PND30. (**b**) Histograms (**left**) and photomicrograph (**right**) representing the number of tertiary branches of mammary glands in control (CON) and treated (GEN) female CD1 mice at PND60 (expressed as mean ± SEM). Scale bar = 0.5 mm.

Mammary gland length gradually increased with development with no significant differences between the experimental groups (Table S1, Supplementary Materials) (Figure 2).

Terminal end buds (TEBs) in virgin mice are present only at pubertal age when they are stimulated by endogenous estrogens and other factors [54]. Indeed, TEBs were present in control animals at PND22 and PND30, while, as expected [55], control mammary glands (at PND60) did not have TEBs. GEN treatment did not affect TEBs at any of the developmental stages considered (Table S1, Supplementary Materials) (Figure 2a).

Moreover, the treatment did not affect the overall architecture of the adult mammary gland. In fact, at PND60, the number of branches was similar in F-CON (6.67 ± 0.22) and F-GEN (7.17 ± 0.51; *p* = 0.408). However, GEN-treated females tended to have mammary glands with a higher score of tertiary branches (F-CON = 1.25 ± 0.48; F-GEN = 3.25 ± 1.11, *p* = 0.17), which develop cyclically during diestrus (Figure 2b).

### 2.2. Male Reproductive Parameters

#### 2.2.1. Testicle Weight

Testicle weight was similar at PND22 (*t*-test; *p* = 1) and PND30 (*p* = 0.207), but in adults, it was significantly lower in M-GEN than M-CON (*p* = 0.05) (Figure 1e). The analyses using two-way ANOVA for age and treatment as independent variables demonstrated a significant difference only for age (F = 67.472; *p* = 0.001), but not for treatment (F = 0.015; *p* = 0.904) and their interaction (F = 2.611, *p* = 0.090).

#### 2.2.2. Testosterone Level

Control males had higher testosterone levels at PND60 (CON vs. PND30 CON; *p* < 0.001) while GEN-treated mice did not show this increase, resulting in significantly lower levels than in control PND60 (*p* = 0.003) (Figure 1f, Table S2).

### 2.3. Leptin

**Figure 3:**
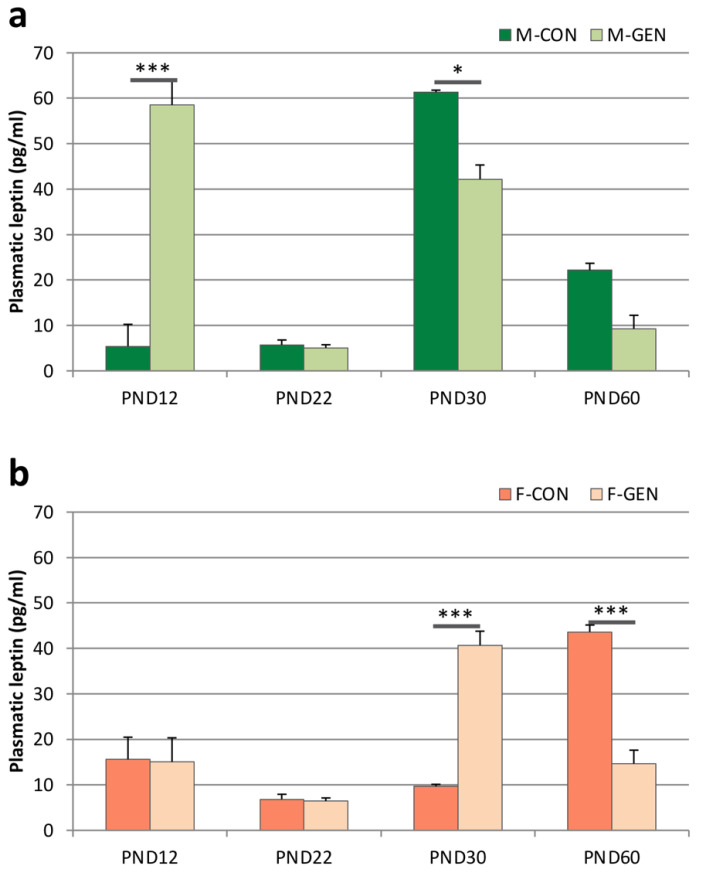
Leptin levels. Histograms representing variations of circulating leptin levels (expressed in pg/mL; mean ± SEM) in males (**a**) and females (**b**), control (CON) and treated (GEN), during the development at postnatal day (PND) 12, PND22, PND30, and PND60. * *p* < 0.05; *** *p* ≤ 0.001 (Tukey test).

In control animals of both sexes, plasma concentration of leptin was low and with no sex differences until PND22 (Figure 3). In females, leptin levels significantly increased at PND60 (PND60 vs. PND30; *p* < 0.001) (Figure 3b). On the contrary, in control males, leptin levels significantly increased at PND30 (PND30 vs. PND22; *p* = 0.001) then decreased at PND60 (PND60 vs. PND30; *p* < 0.001) (Figure 3a). An interesting dimorphism was present at PND30 when males showed a strong peak of plasma leptin (M-CON vs. F-CON; *p* = 0.014), while at PND60 the concentration was significantly higher in females compared to males (M-CON vs. F-CON; *p* = 0.008).

This dimorphism was abolished by GEN treatment. In fact, in males, GEN treatment induces an early significant increase in leptin level at PND12 in comparison to control animals (M-GEN vs. M-CON; *p* < 0.001). Plasma concentration of leptin reached a peak at PND30 in both GEN experimental groups, although it was significantly lower in GEN treated males than in CON (M-GEN vs. M-CON; *p* = 0.032). No significant difference was observed at PND60 (M-GEN vs. M-CON; *p* = 0.456; Figure 3a). A similar trend was observed in the GEN-treated females, where changes in circulating leptin were similar to M-GEN and M-CON, with a peak at PND30 and a decrease at PND60, both significant in comparison to F-CON (*p* < 0.001; Figure 3b). Plasma concentrations of leptin in treated males were significantly higher than F-GEN only at PND12 (M-GEN vs. F-GEN; *p* < 0.001).

### 2.4. Kisspeptin System

The pattern of distribution, development, and dimorphism of kiss-ir structures observed within the hypothalamic region of control animals was consistent with previous observations in rodents [24,25]. The quantitative analysis of CON and GEN groups suggested that postnatal exposure to GEN significantly altered the development of kisspeptin expression in the hypothalamic nuclei under study in a dimorphic manner (all data are reported in Supplementary Materials, Table S3).

**Figure 4:**
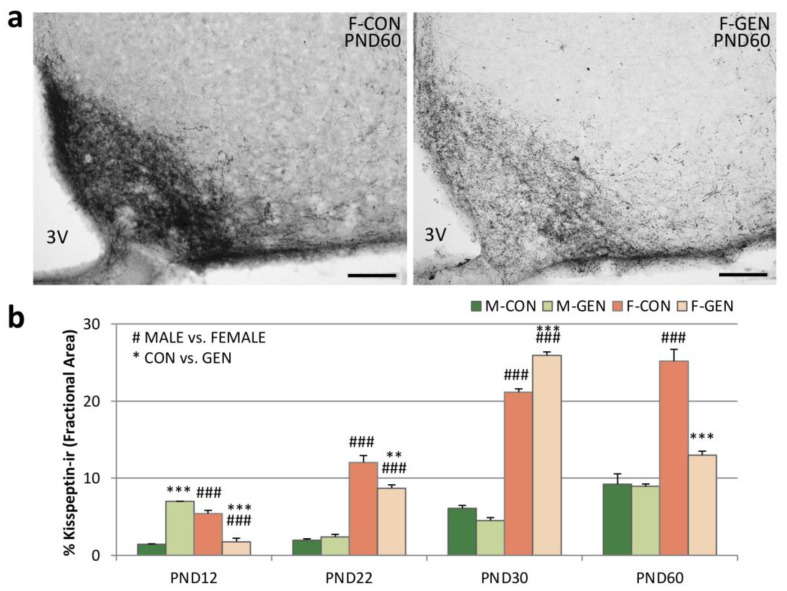
Kisspeptin immunohistochemistry. (**a**) Photomicrographs showing kisspeptin immunostaining in the ARC in adult (PND60) CD1 control (F-CON) and treated (F-GEN) female mice at PND60. 3V, third ventricle. Scale bar = 100 µm. (**b**) Histograms representing the variations of the percentage of area (FA; mean ± SEM) covered by kisspeptin immunopositive elements in the ARC of both sexes (M-CON, M-GEN, F-CON, and F-GEN) at different ages (PND2, PND22, PND30, and PND60). ** *p* ≤ 0.01; *** *p* ≤ 0.001; ### *p* ≤ 0.001 (Bonferroni test).

In the ARC, the high density of kiss-ir precluded our ability to distinguish cell bodies (Figure 4a); therefore, we quantified the fractional area (FA). Early postnatal exposure to GEN deeply influenced the expression of the kiss in the ARC. In males, GEN influenced kiss expression only at PND12, when M-GEN showed a significantly higher expression of kiss, which was also higher than in females (M-GEN vs. F-GEN; *p* < 0.001), reversing the sex dimorphism of the system (Figure 4b). No difference was detected in the comparison M-CON versus M-GEN at PND22, PND30, and PND60. In females, the situation was different: at PND12 (*p* < 0.001) and at PND22 the FA in F-GEN remained significantly lower in comparison to F-CON (*p* = 0.004), but after puberty, at PND30, the kiss-ir signal increased in treated females (*p* < 0.001). Then, at PND60 FA decreased in F-GEN animals (Figure 4a). GEN treatment seems to cause anticipation of the development of the system in females at PND30 (Figure 4b).

**Figure 5:**
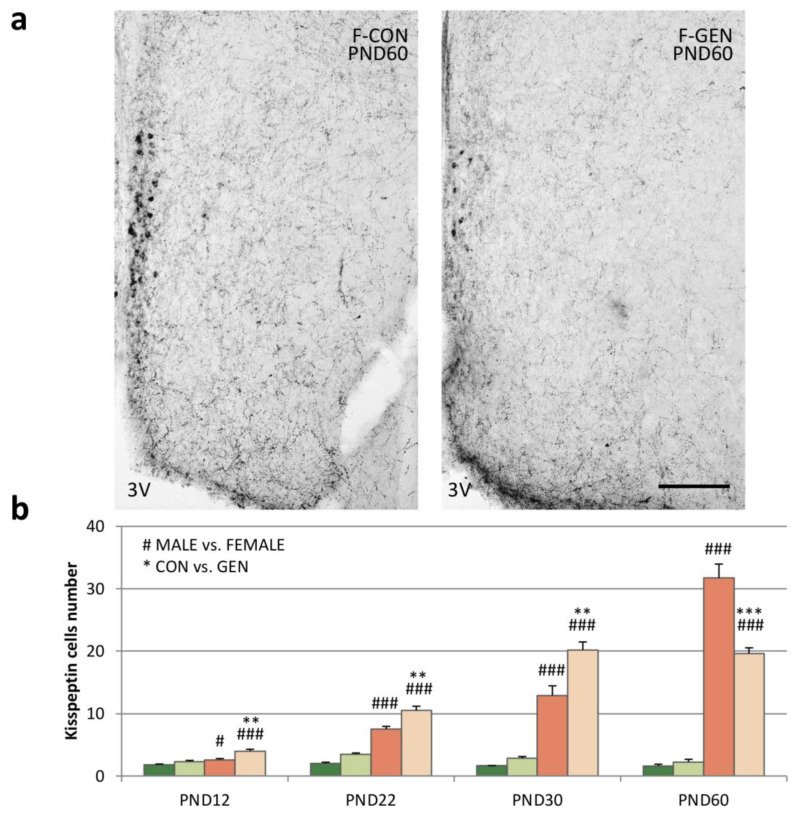
Kisspeptin immunoreactivity. (**a**) Photomicrographs showing kisspeptin immunostaining in the RP3V of adult (PND60) control (F-CON) and treated (F-GEN) CD1 female mice. 3V, third ventricle. Scale bar = 100 µm. (**b**) Number of kisspeptin-positive cells (mean ± SEM) in the RP3V of both sexes (M-CON, M-GEN, F-CON, and F-GEN) at different ages (PND12, PND22, PND30, and PND60). ** *p* ≤ 0.01; *** *p* ≤ 0.001; # *p* ≤ 0.05; ### *p* ≤ 0.001 (Bonferroni test).

Within RP3V we analyzed the number of kiss-ir cells. In control animals, the system was dimorphic at every age, with a higher number of cells in females. In addition, while the number of cells was stable for all the considered ages in males, there was a significant increase in females, from PND12 to PND60. Early GEN postnatal treatment had no significant effect on males at any of the considered ages (Figure 5b), while females were significantly affected. The number of cells was significantly higher in F-GEN at PND12 (*p* = 0.002), PND22 (*p* = 0.002), and PND30 (*p* = 0.003) in comparison to F-CON. At PND60 the number of cells in the F-GEN group was close to PND30. Thus, at PND60 the F-CON showed a higher number of positive cells than F-GEN (*p* < 0.001) (Figure 5a,b). Therefore, it appears that kiss-ir cells reached a plateau at PND30 in F-GEN, whereas this peak is reached only at PND60 in F-CON.

**Figure 6:**
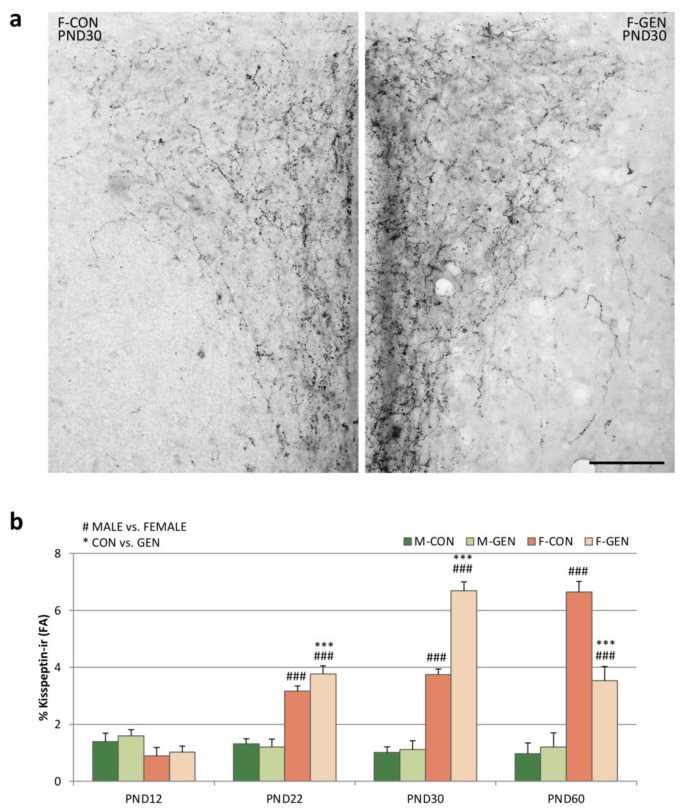
Kisspeptin innervation of the PVN. (**a**) Comparison of the PVN innervation at PND30, when the F-GEN group has its peak. (**b**) Variations of the fractional area (FA; mean ± SEM) covered by kisspeptin immunoreactive fibers in the PVN of both sexes (M-CON, M-GEN, F-CON, and F-GEN) at different ages (PND12, PND22, PND30, and PND60). Scale bar = 100 µm. *** *p* ≤ 0.001; ### *p* ≤ 0.001 (Bonferroni test).

In the PVN (Figure 6a), the effect of GEN treatment followed more that observed in RP3V than in the ARC. We observed no effects in males at all the considered ages, whereas in females we observed a precocious increase of kiss-ir in F-GEN animals at PND22 and PND30, with a sharp decrease at PND60. As for the ARC and RP3V in F-CON, the kiss-ir increased in the PVN, reaching the highest value at PND60 (Figure 6b).

**Figure 7:**
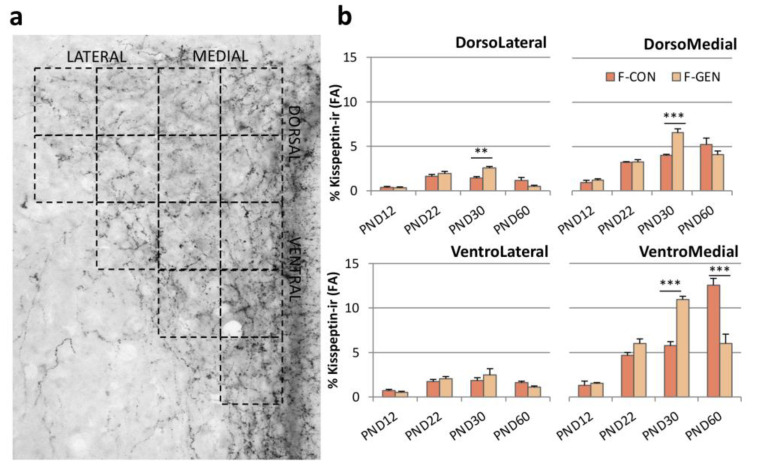
(**a**) Photomicrograph of kisspeptin immunostaining in the PVN of adult control females, showing the subdivision of the PVN in fourteen quadrants to identify the four parts of the nucleus (DM, dorso-medial; DL, dorsolateral; VM, ventro-medial; VL, ventro-lateral). (**b**) Variations of the FA covered by kisspeptin immunoreactive fibers in the different parts of the PVN (DM, DL, VM, VL) in control (F-CON) and treated (F-GEN) female CD1 mice at different ages of sacrifice (PND12, PND22, PND30, and PND60). Scale bar = 50 µm. ** *p* ≤ 0.01; *** *p* ≤ 0.001 (Bonferroni test).

The distribution of kiss-ir fibers within the PVN, as also reported in previous studies [24,56], was not homogeneous but was denser in the medial versus lateral PVN (Figure 7a). In M-CON, kiss-ir was similar in the different parts of the PVN, although it tended to be higher in the ventro-medial part. No significant differences were observed at any age or after GEN treatment. On the other hand, in females (Figure 7b), the GEN effect was evident within dorso- and ventral-medial parts, where the kiss-ir fibers were denser. F-GEN reached the highest value of FA in the DM and VM part of the PVN at PND30, with a decrease at PND60, whereas the F-CON reached the highest value at PND60.

### 2.5. Metabolic Parameters

#### 2.5.1. Body Weight, Food Consumption, and Feed Efficiency (FE)

The analysis using three-way ANOVA of body weight measured, with age, sex, and treatment considered as independent variables, showed a significant effect both of the interaction sex and treatment (F = 8.90; *p* = 0.003) and sex and age (F = 34.735; *p* = 0.001), but not for the interaction treatment and age (F = 1.806; *p* = 0.079) or among all independent variables (sex * age * treatment; F = 0.698; *p* = 0.693). We did not observe any effect of GEN on the metabolic parameters considered during and after the treatment until day 30. Starting from PND30, the body weight presented strong differences among experimental groups (Table S4, Supplementary Materials). The analysis via Tukey’s test for M-CON vs. F-CON reported a significantly higher weight in males (PND30, PND40, PND50, and PND60, *p* < 0.001).

**Figure 8:**
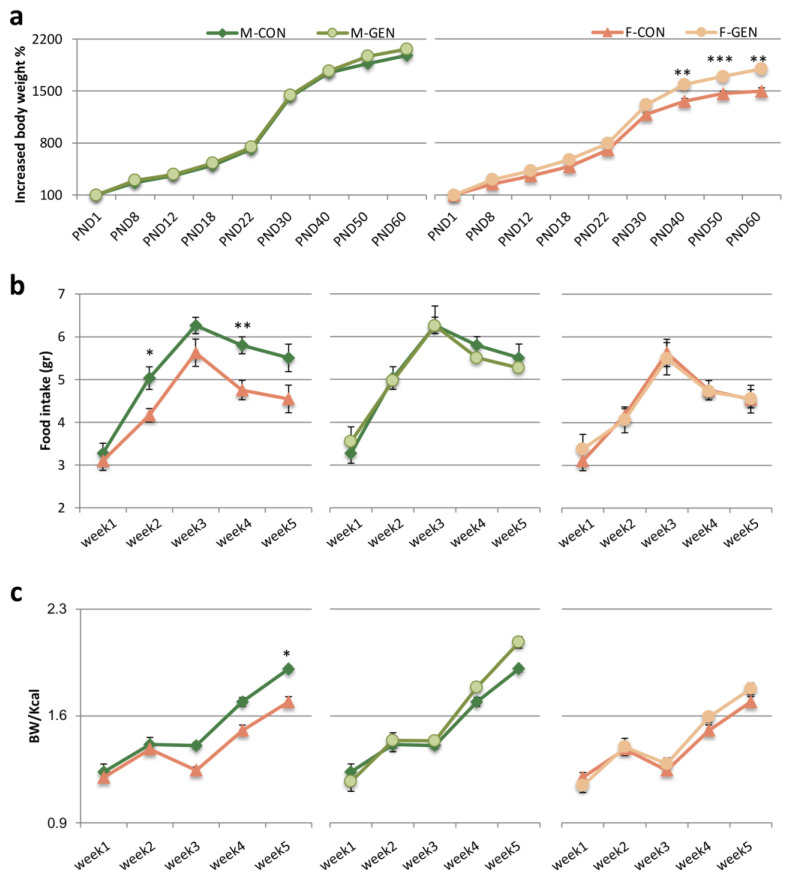
Metabolic parameters. (**a**) Lines representing the increase of body weight in percentage, with the weight at PND1 = 100 (mean ± SEM) during the development of male control (M-CON) vs. male-treated (M-GEN), on the left, and female control (F-CON) vs. female-treated (F-GEN), on the right. (**b**) Variations of the amount of food intake expressed in grams (mean ± SEM) during five weeks after weaning in both sexes (M-CON, M-GEN, F-CON, F-GEN). On the left a comparison among control males and females; the other two diagrams illustrate the comparison within each sex among control and genistein-treated mice. (**c**) Variations of the daily feed efficiency (body weight/kcal introduced; mean ± SEM) calculated during five weeks after weaning in both sexes (M-CON, M-GEN, F-CON, F-GEN). On the left a comparison among control males and females; the other two diagrams illustrate the comparison within each sex among control and genistein-treated mice. * *p* < 0.05; ** *p* ≤ 0.01; *** *p* ≤ 0.001; (Bonferroni test).

Differences were observed also in the percentage of body weight gained since PND30 (Figure 6a). Two-way ANOVA for sex and treatment confirmed this result (respectively F = 15.39; *p* = 0.001 and F = 5.901; *p* = 0.025). No differences were present between controls and treated males, while a significant increase was observed starting from PND40 in F-GEN compared to control females (Tukey test; F-CON vs. F-GEN at PND40 *p* = 0.007; at PND50 *p* = 0.001; at PND60 *p* = 0.005) (Figure 8a).

Daily food consumption per animal during the weeks after weaning was calculated as reported in Materials and Methods. Generally, we observed lower food consumption in F-CON in comparison to M-CON. This trend was observed in GEN-treated animals with males treated with GEN eating significantly more food than F-GEN only during the second (*p* = 0.038) and fourth (*p* = 0.011) week (Table S5, Supplementary Materials) (Figure 8b). However, no differences were found between control and treated animals either in males or in females. The two-way ANOVA for repeated measures (independent variables: sex and treatment, repeated measure: weekly food consumption) showed, in fact, only an effect of sex (F = 21.015 and *p* = 0.001), with no effect of treatment (F = 0.165 and *p* = 0.689) and no significant interaction between sex and treatment (F = 0.056 and *p* = 0.815).

Feed efficiency (body weight gain/Kcal) was analyzed using two-way ANOVA for repeated measures (independent variables: sex and treatment, repeated measure: feed efficiency) showing a global effect of sex (F = 6.722 and *p* = 0.0017), but no effect of treatment (F = 0.704 and *p* = 0.411). The multiple comparisons between groups displayed a sexual dimorphism in controls, with a significant increase in M-CON vs. F-CON in the last week of the experiment (from PND53 to PND60; Tukey test, *p* = 0.038). This difference was present also between M-GEN and F-GEN at week 3 (*p* = 0.05) and week 4 (*p* = 0.03; Figure 8c). GEN treatment did not alter feed efficiency either in males or females (Table S6).

#### 2.5.2. Triiodothyronine (T3) and Ghrelin Levels

Plasma concentration of T3 at PND30 showed no significant differences. In the adult animals, T3 levels were significantly higher in females than in males (F-CON vs. M-CON; *p* = 0.001). GEN treatment did not affect T3 levels in males (M-CON vs. M-GEN; *p* = 1), but it reduced the plasma concentration of T3 levels in adult females at PND60 close to significant (F-CON vs. F-GEN; *p* = 0.05), abolishing sex dimorphism (F-GEN vs. M-GEN; *p* = 1; Table 1).

The analysis of plasma concentration of ghrelin at PND30 did not present significant differences among the experimental groups. In adults, at PND60, F-CON animals showed a higher plasma concentration of hormone in comparison to males (F-CON vs. M-CON; *p* = 0.001). The postnatal treatment with genistein completely reversed this situation, the sex difference disappeared (F-GEN vs. M-GEN; *p* = 0.146): the plasma concentration of ghrelin significantly increased in GEN males (M-CON vs. M-GEN; *p* = 0.001) and decreased in GEN females, although not significantly (F-CON vs. F-GEN; *p* = 0.06), compared to control animals (Table 1).

### 2.6. Hypothalamic Systems Controlling Food-Intake

#### 2.6.1. POMC System

**Figure 9:**
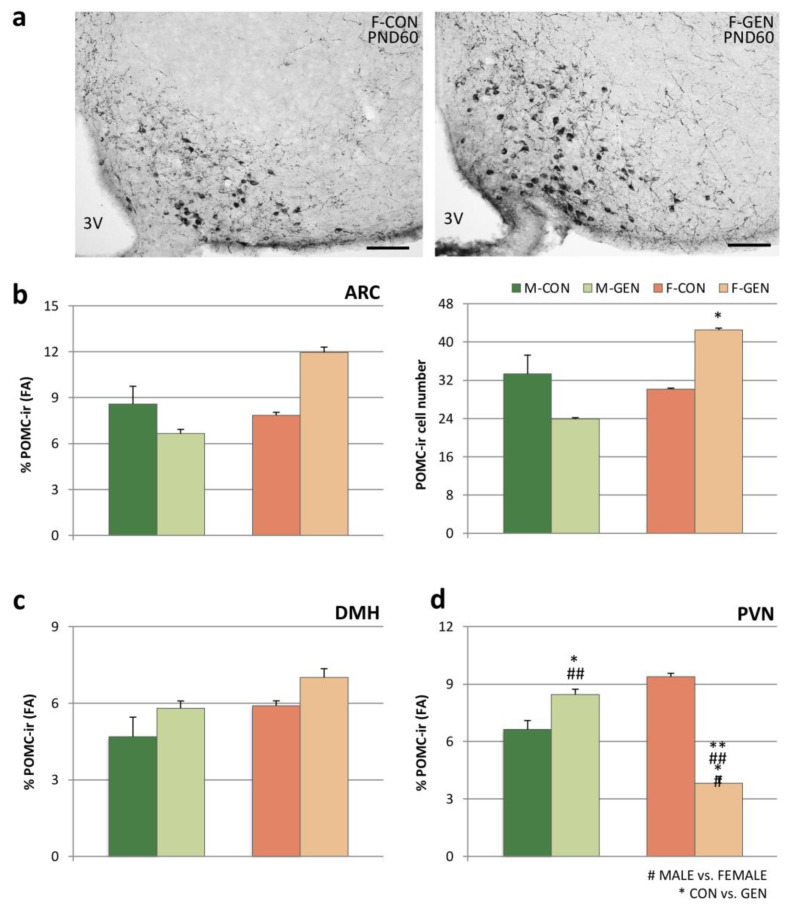
Pro-opiomelanocortin (POMC) immunoreactivity. (**a**) Photomicrographs of the arcuate nucleus (ARC) immunostained for POMC at PND60 in control (F-CON) and treated (F-GEN) female mice. 3V, third ventricle. Scale bar = 100 µm. (**b**) Variations of both the percentage of area (FA: mean ± SEM) covered by POMC immunopositive structures (left) and the number of positive cell bodies (right) in the ARC of both sexes (M-CON, M-GEN, F-CON, F-GEN) for adult mice. (**c**,**d**) Histograms representing the FA (mean ± SEM) covered by POMC immunoreactivity within (**c**) the dorsomedial hypothalamic nucleus (DMH) and (**d**) paraventricular nucleus (PVN). * *p* < 0.05; ## *p* ≤ 0.01 (Bonferroni test).

According to our previous study [57], in the present experiment, the POMC-ir in the ARC was very similar in adult mice of both sexes (Table S7). In fact, the Bonferroni test did not show any significant difference in POMC-ir cell number (*p* = 0.896) or in FA covered by ir structures (*p* = 0.918) in control animals. GEN treatment significantly affects the adult profile of POMC expression in the ARC in a sex-specific manner. Two-way ANOVA reported a significant effect on the sex and treatment interaction (cells number, F = 13.710; *p* = 0.002; FA, F = 7.371; *p* = 0.015). Indeed, the alterations of the POMC-ir elements were limited to females (Figure 9a). The Bonferroni post hoc test showed statistically significant higher values in F-GEN than F-CON for the number of POMC cells (*p* = 0.047), but not for FA (*p* = 0.081) in the ARC, while no difference was present in males (*p* = 0.657 and *p* = 0.134, respectively for FA and cellular number) (Figure 9b; full quantitative data are in Table S7 for adults and in Table S8 for the development).

The immunoreactivity of POMC fibers within DMH did not result in significant differences both in controls and in GEN-treated animals (Figure 9c).

POMC-ir was dimorphic in the PVN of control animals: Bonferroni test showed that the FA covered by ir structures (in this case only fibers) was higher in females (*p* = 0.002). The two-way ANOVA demonstrated a significant effect of sex (F = 5.455; *p* = 0.036), of treatment (F = 21.667; *p* < 0.001), and of the interaction sex/treatment (F = 84.642; *p* < 0.001). The early postnatal GEN exposure decreased the immunoreactivity in the female PVN (*p* < 0.001) and increased it in males (*p* = 0.048), with a completely dimorphic effect. In fact, in GEN animals the dimorphism was inverted, higher in males than in females (*p* < 0.001; Figure 9d). This effect was predominant in the medial part of the PVN, where the FA strongly decreased in treated females (DM, *p* < 0.001; VM, *p* < 0.001).

#### 2.6.2. Orexin System

**Figure 10:**
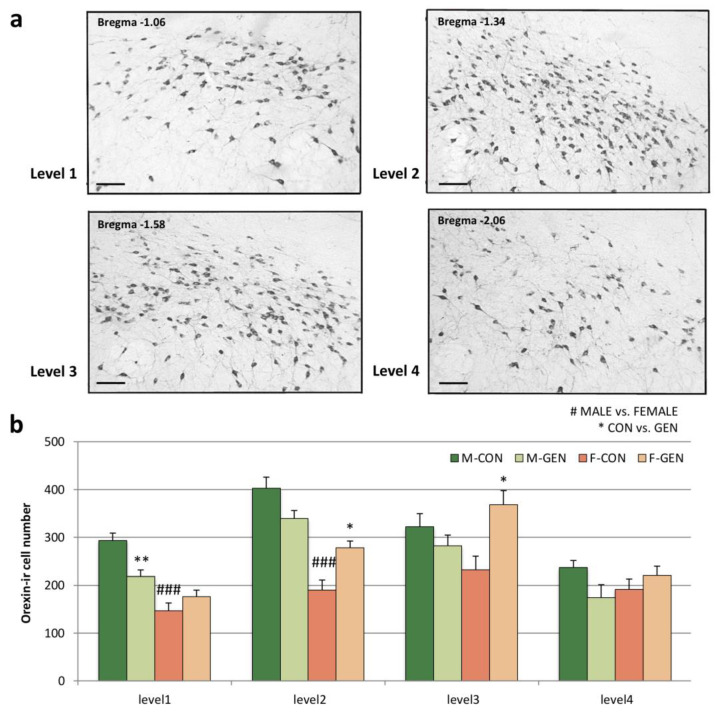
Distribution of orexin cells within the lateral hypothalamic nucleus. (**a**) Photomicrographs showing the distribution of orexin-positive cells in four different rostro-caudal levels of the lateral hypothalamus (LH), corresponding to Bregma −1.06, −1.34, −1.58, and −2.06 of the Mouse Brain Atlas [59] in male control CD1 mice at postnatal day (PND) 60. Scale bar = 100 µm. (**b**) Variations in the number of orexin-positive cells (mean ± SEM) in different levels of the LH of adult CD1 mice of both sexes (M-CON, M-GEN, F-CON, F-GEN). * *p* < 0.05; ** *p* ≤ 0.01; ### *p* ≤ 0.001 (Bonferroni test).

As reported in previous studies (for a review see [58]), in both sexes of adult CD1 mice, the lateral hypothalamus (LH), in its full rostro-caudal extension (Figure 10a), contains a large number of orexin-ir cells. Considering the total number of positive cells, the two-way ANOVA for sex and treatment showed a significant effect of treatment (F = 17.502; *p* = 0.001). In controls, the system is sexually dimorphic with males having a significantly higher number of cells (313.7 ± 18.21) than females (189.9 ± 19.29, Bonferroni test M-CON vs. F-CON; *p* < 0.001). Post hoc Bonferroni showed a statistically significant decrease of cell number in GEN-treated male mice (253.4 ± 14.70) in comparison to M-CON (*p* = 0.041), while in females (260.7 ± 10.90) a significant increase of cell number was present in F-GEN vs. F-CON (*p* < 0.05). Because of the different effects in males and females, the dimorphism completely disappeared in GEN-treated mice (Bonferroni test, M-GEN vs. F-GEN; *p* = 1) (adult data are in Table S9A and changes during the development are reported in Table S8).

Considering the distribution of orexin-positive cells along the rostro-caudal axis (Figure 10a), the two-way ANOVA revealed a significant difference for level and a significant interaction for level and sex (respectively F = 43.843 *p* < 0.001; F = 17.727 *p* < 0.001). The two-by-two comparison (Bonferroni test) showed a significant decrease in the anterior region of M-GEN (M-GEN vs. M-CON; level1 *p* = 0.013), while in females the cell number increased in the more caudal region of the LH (F-GEN vs. F-CON level2 *p* = 0.04 and level3 *p* = 0.025) (Figure 10b, full data are in Table S9A).

In the PVN of control animals, the OX system was not dimorphic via comparison with the Bonferroni test (*p* = 0.8). Two-way ANOVA showed a significant effect of the interaction sex/treatment in adult animals within the PVN (F = 16.09; *p* = 0.002). The early postnatal GEN treatment significantly decreased the OX-ir fibers in males (*p* = 0.045), while they slightly increased in females (not significantly; *p* = 0.088). Interestingly, this sexual dimorphism was more pronounced in treated animals, in which the FA was higher in GEN females than in GEN males (*p* = 0.009), especially in the ventromedial part of the nucleus (*p* = 0.002) (Table S9B).

## 3. Discussion

Genistein, an isoflavone contained in soy, has an estrogen-like structure and exerts its effects by binding to the estrogen receptors: *in vitro* studies demonstrated that ERα, ERβ, and the estrogen membrane receptor (GPR30) are important for the neuritogenic effect of GEN [12]. The impact of GEN as an endocrine disrupting chemical (EDC) on health is still debated [37]. GEN alters estrous cyclicity, fecundity, ovulation, and female reproductive behavior [60,61], and when the exposure occurs at neonatal age, even at environmentally relevant doses, it affects female development and persists into adulthood [62]. In the present experiment, we observed precocious vaginal opening (an index of precocious female puberty [53]), an increase of uterus weight at pre-puberal age, irregular estrous cycles, with a reduction of the proestrus phase, probably correlated to an increase of plasma concentration of progesterone, and elongation of the diestrus phase.

In addition, we also analyzed the mammary gland, another important female reproductive target of EDCs. The analysis reported that the mammary glands were not directly affected by postnatal GEN treatment. This was not surprising since early development and sexual differentiation of the mammary gland occur before birth, thus perturbations of mammary gland development by EDCs have been observed only when the exposure was during the gestational period (i.e., GEN [63] or BPA [64]). The allometric postnatal growth of the mammary gland is independent of sexual steroids [55], while it responds to endocrine stimulation from puberty [55]. We observed that the number of animals displaying TEB at PND22 was higher in the GEN-treated group than in the controls, even if the difference was not significant. Since branching morphogenesis initiates at puberty [55], these data confirm that puberty is premature in GEN-treated animals. In addition, post-weaning GEN treatment induced advanced puberty, and this treatment had a more apparent effect on the mammary gland anatomy [65].

All these peripheral effects could reflect the alterations of the kiss system described in the same animals. Many studies demonstrated that the kiss is an important target for EDCs action (i.e., bisphenol A [66]) but very limited data are available on the impact of phytoestrogens on kisspeptin circuits in mice. In female rats, neonatal exposure to GEN (10 mg/kg) induced a persistent masculinization of kiss-ir fibers in RP3V (lower density), but not in the ARC [11], while developmental estrogen exposure significantly decreased kiss immunostaining in the ARC [11]. Moreover, postnatal GEN exposure does not significantly affect kiss-ir levels in adult males [49]. Consistently, we observed that early postnatal exposure to GEN induced sexually dimorphic effects on the kiss system. In males, GEN induced only a transient increase in the kiss-ir FA in the ARC at PND12. Interestingly, this increase was concomitant to a significant peak of plasma concentration of leptin, which is considered a positive modulator of the kiss system [29,67]; in fact, the energy balance is undoubtedly strictly correlated to the reproductive function. In our animals, the same correlation was also present in females, but at a post-pubertal age. The observed early increase of kiss immunoreactivity in the ARC, RP3V, and PVN correlated with a peak of plasma concentration of leptin measured in PND30 females treated with GEN.

Not many studies have focused on the possibility that a soy-based diet could alter circulating leptin levels. However, from the literature, the leptin concentrations in serum were affected by GEN, but the effect depends on sex, age, and the hormonal status in which the treatment was carried out (as reviewed in [68]). Unsurprisingly, we also observed here a strong variability in serum leptin levels during the development of treated mice based on both sex and age. Nevertheless, a decrease in plasma levels of leptin levels occurred in both sexes in adulthood, but especially in GEN females. The hipoleptinemic action of GEN was documented in male adult rats, showing a reduction in leptin levels after only three days of treatment with a dose of 5 mg/kg body weight [69]. In female rats, GEN has no effects on the plasma levels of normal females, but it induces a reduction of serum leptin in pregnant females [70], as well as in obesity models [71,72].

Negative effects of EDCs on reproduction are well described, but, in recent years, some EDCs have been considered among the multiple environmental factors that have been linked to the increase in obesity and metabolic syndrome, and they were named metabolic disrupting chemicals (MDCs) [73]. These MDCs can act indirectly to promote adipogenesis and cause weight gain by shifting energy balance to promote calories accumulation, altering basal metabolism [74], and altering hormonal control of appetite and satiety [8]. More recently, GEN has also been included in the list of MDCs [37], and these effects emerged strongly in our work. Note that susceptibility to obesity begins during development (in utero and early life), critical periods in which MDCs can influence developmental planning and thus disrupt the set point for weight gain later in life [75]. Contradictory data exist, underlining the importance of exposure times, dose/concentration, and sex to establish safety recommendations for the intake of GEN in the diet, especially if in early life. The same treatment may have a different outcome depending on the age and the sex of the animal. Compared to dams on a soy-free diet, rats on a soy-enriched diet gain less weight during pregnancy, and although they consume more food, they do not become heavier during lactation. Their offspring, however, are significantly heavier (both sexes, but more pronounced in males), show higher food intake, and females have an earlier pubertal onset [39]. GEN postnatal oral administration (PND1 to PND22) of 50mg/kg GEN (the same dose as our study) in rat pups resulted in a similar effect in females and increases adipocyte size and number, fat mass, and fat/lean mass ratio and decreased the size of muscle fiber [45]. Consistently, in a previous study [14] we demonstrated an obesogenic effect of postnatal GEN (administrated from PND1 to PND8) in adult female CD1 mice only. Here, we confirmed this effect, from puberty until adulthood. The increase in body weight was not correlated to alterations of food intake and daily feed efficiency, indicating a probable metabolic disruption. Concurrently, only in GEN-treated females, plasma concentration of two important metabolic hormones, leptin and T3, were significantly decreased, while the serum ghrelin showed a strong increase in GEN males only.

Indeed, GEN induces similar metabolic changes as well as alterations in the T3, ghrelin, and leptin in other models [68]. In fact, this increased plasma concentration of T3 only in female mice has also been shown in NIH/S female mice postnatally exposed to GEN (8 mg/kg body weight/day) [76] and in the golden Syrian hamster exposed to a soy protein diet for 28 days [77]. GEN also affected in a sexually dimorphic way the ghrelin concentration, with a decrease in females, as was observed in previous studies in mice [76]. In addition, in other experimental models (i.e., Mustela family) in adult females, there was a reduction in levels of plasma concentration of ghrelin, although not fully significant [78], while a strong increase was observed in males [79]. The reported alterations in plasma concentrations of the analyzed metabolites, associated with increase of weight gain only in female mice treated with GEN, without any alteration in the amount of ingested food, suggest the control of energy expenditure is susceptible to postnatal treatment with genistein in a sexually dimorphic way.

In addition to the previously discussed direct (or indirect) effects on the kiss system, leptin decrease in the adult may, in turn, impair the activation of the hypothalamic POMC system. POMC neurons in the ARC have an anorexigenic action on food-intake control. These neurons co-express different neuropeptides and a wide variety of receptors, including leptin receptors [80]. Moreover, the POMC system is sensitive to gonadal steroids [81], and we recently demonstrated that this system could be a good target for MDCs action by using a chronic exposure to tributyltin [57], as well as long-term exposure to BPA, DES, and TBT [82]. Here, we confirm that also early postnatal exposure to GEN induces long-term sex-specific organizational effects on the POMC system in the ARC and PVN.

Another hypothalamic system controlling metabolism and food intake is the OX system. This peptide modulates energy balance based on food intake by discriminating the physiological variation of glucose levels between meals [18]. Moreover, OX enhances spontaneous physical activity and regulates energy expenditure thus promoting obesity resistance [83]. In adult mice, the number of OX neurons in the LH is higher in males than in females [84]. This dimorphism could be associated with sexual male maturation, since in adult male rodents, OX neurons are markedly activated during copulation [85]. Interestingly, we observed that this dimorphism, observed in our control groups, was totally reverted in GEN mice: GEN increased the cell number in females while decreased it in males. OX neurons co-express a few estrogen (ER-alpha) receptors and no androgen (AR) receptors [85]; therefore, its sex dimorphism may be due to indirect control by gonadal steroids, and early exposure to GEN may, thus, permanently interfere with OX system differentiation. Moreover, we demonstrated that different rostrocaudal levels of the LH harbor OX subpopulations with specific features. In fact, males had more OX-ir cells than females in the most rostral levels, while females presented a higher number of OX cells in the most caudal levels. Furthermore, those subpopulations displayed a sexually dimorphic response to GEN postnatal treatment, with a decrease in the number of OX cells in adult males in rostral levels of the LH and an increase in more caudal levels of females. Future studies should investigate if these different OX subpopulations, located in specific rostrocaudal domains of the LH, have different targets and roles.

Our hypothesis that postnatal treatment with GEN may act on the control of energy expenditure is supported by the observed alterations of all the analyzed systems projecting to the PVN. In fact, the PVN is the most important hypothalamic center of metabolic control, modulating feeding behavior through the action of CRH and TRH, both indirectly via effects on energy expenditure (hypothalamus-pituitary-thyroid axis, HPT) and directly through the HPA axis [23,86].

As demonstrated in previous studies [24,56], the PVN is rich in kiss fibers, especially in the medial part of the nucleus, where CRH and TRH neurons are located, suggesting a strong correlation between reproductive and metabolic control [24]. In fact, PVN kiss fibers are part of a sexual network essential for the control of the HPG axis through the control of the GnRH system. The PVN is also rich in NPY and POMC fibers [82], as well as in orexin fibers (present study).

Cell numbers in the ARC and their projections may respond differently to treatment with EDCs, which is particularly evident for the POMC system [57,82]. Present results show a reduction of POMC innervation of the PVN in females and an increase in males, while cell numbers in the ARC have an opposite trend. This could be due to the presence of subpopulations of POMC cells in the ARC expressing different receptors [34,80,81,87] and that potentially send their projections to different targets. In addition, a potential direct effect of kisspeptin on POMC neurons also cannot be ruled out. In fact, kisspeptin in the ARC directly excites POMC neurons [33] that express kiss1 receptors [34]. Both systems are affected by GEN treatments in our animals, and this could also have a differential impact on their projections.

As previously discussed, OX regulates food intake and energy homeostasis, but also reproduction. Orexin has in fact a direct impact on GnRH [88] and kiss [89] neurons. Moreover, OX receptors have also been found in rat testes, and testosterone secretion is directly stimulated by OX [90]. In a previous experiment, we have shown, in males postnatally exposed to GEN, a decrease in the ratio prostate/BW and to a lesser extent in the ratio testis/BW [14]. However, in this experiment, we have observed a decrease of fecal testosterone and of the weight of testicles, and we also observed a decrease in the number of OX neurons in LH, suggesting that OX may act at the testicular level also in mice. Furthermore, a previous study has shown that exposure to GEN (8 mg/kg body weight/day) early in life leads to changes in the reproductive organs of males, with relative weights of the prostate and seminal vesicles being greater than in control males. [76].

## 4. Materials and Methods

### 4.1. Animals

We purchased from Charles River, France, 26 female and 13 male adult virgin CD-1 mice. All experiments were performed according to the EU directive on animal experimentation 2010/63 and approved by the local ethical committee (Comité d’Ethique en Expérimentation Animale Centre-Val de Loire) under approval 426-201504031706655.

Animals were housed in monosexual groups of 3 mice in conventional polycarbonate cages (45 × 25 × 15 cm) with water and food (standard diet 150 low phytoestrogen certificate, SAFE, France) ad libitum and exposed to a 12-h light/dark cycle. After 2 weeks of the adaptation period, females were housed with males in groups of 2 females and 1 male for one night, beginning at 18:00 h (at the end of the light phase); after the mating, verified by the presence of a vaginal plug (generally 3–5 days), the females were placed in single cages.

### 4.2. Genistein Treatment

The day after the birth, postnatal day one (PND1), litters were reduced to 8 pups, 4 males and 4 females, sexed via anogenital distance [91]. Pups were then allocated randomly to two groups and subjected from PDN1 to PND8 to oral administration of vehicle (10 μL/g sesame oil) or genistein (GEN 50 mg/kg body weight; cat. Number G6649, Sigma-Aldrich, St. Quentin Fallavier Cedex, France) diluted in sesame oil. This protocol mimics the exposure of babies fed with soy-based formulas [92]. Moreover, as previously shown in a pharmacokinetic study, the dose we used produces serum levels of GEN in neonatal mice, within the human range [93]. Mice spontaneously drank the solution through a micropipette directly into the mouth [14,15]. Three weeks after birth (PND21), the pups were weaned and housed in single-sex groups of 3–5 animals, differentiated by treatment, in polypropylene mouse cages.

Animals were divided into 4 groups: control males (M-CON), control females (F-CON), genistein-treated males (M-GEN), and genistein-treated females (F-GEN), and sacrificed at PND12, PND22, PND30, and PND60. Six mice per group were perfused for immunohistochemical studies of the neuronal circuits, while the others were killed by decapitation (N = 6 per group) to study the peripheral parameters.

### 4.3. Reproductive and Metabolic Parameters

#### 4.3.1. Vaginal Opening and Estrous Cycle

After weaning, GEN-treated and control females (N = 24 per group of treatment) were checked daily, from PND19 to PND29, for vaginal opening (VO) to detect the time of puberty [53].

From PND40 to PND55 daily microscopic inspection of vaginal smears flushed with physiological saline solution was performed in F-CON (N = 6) and F-GEN (N = 6) to determine the phase of the estrous cycle. The percentage of the days in each phase was calculated.

#### 4.3.2. Uterus and Testicle Weight

The weight of reproductive organs, uterus, and testicles, obtained from female (N = 6) and male (N = 6) mice, were manually dissected and measured in each group at PND22, PN30, and PND60 after decapitation. Testicle weight was calculated by summing the weights of the two testicles for each male.

#### 4.3.3. Mammary Gland Analysis

Mammary glands were collected at PND22, PND30, and PND60 from females in the diestrus phase of the cycle (determined by vaginal smear). Briefly, the fourth mammary gland (inguinal) was dissected from the skin, stretched on a glass slide, and fixed in Carnoy’s fixative at 4 °C for 2 h and then stored in the same solution until processing. Whole mounts were gradually re-hydrated, stained with Carmine Alum (Stem Cell Technologies) overnight, disdained for 2 h in 70% EtOH with 2% HCl, progressively dehydrated, clarified in methylsalicilate overnight, and photographed.

Whole mounts were photographed at 1× and 4× on a Leica S8AP0 stereomicroscope equipped with a Leica EC3 digital camera. Mammary gland length was measured with Image J software (version 1.47v; Wayne Rasband, NIH, Bethesda, MD, USA), as the distance between the beginnings of the duct arising from the nipple and the end of the more distal ducts of the glands. The total number of branches was counted at PND30 and PND60 as the number of branches 3.5 mm before and 3.5 mm after the lymph node after it. The number of tertiary branches was scored on a scale ranging from no branches (0) to high density (5).

#### 4.3.4. Body Weight, Food Consumption, and Feed Efficiency (FE)

Body weight was recorded daily during the treatment and every two days from PND8 to sacrifice with an electronic precision balance (Mod. Kern-440-47N). To eliminate differences due to the variability between animals, we normalized the absolute body weight into a percentage of the body weight of the first day of the treatment (PND1), conventionally considered equal to 100.

Animals were fed with a standard diet 150 low phytoestrogen certificate (SAFE, Augy, France) containing 3264 Kcal/g of metabolizable energy with 21% as protein, 12.6% as lipid, and 66.4% as carbohydrate. Mean food consumption (mean grams per mouse per day) was determined every two days at 10.00 a.m. All animals from each group were housed in standard cages (each containing 3 animals). The daily food consumption per animal was estimated by dividing the total food consumption (total amount of food supplied per cage minus the weight of the residual food in each cage) by the number of mice in the cage and the number of days after the last measurement. After the measurement, fresh food was given to the mice. Daily energy intake was calculated by multiplying daily food intake by the caloric value of the chow (3264 Kcal/gr), and daily feed efficiency was expressed as body weight (gr)/Kcal eaten [94].

#### 4.3.5. Hormonal Levels

Blood samples were collected at PND12, PND22, PND30, and PND60 from animals killed by decapitation, which always took place in the morning between 9 AM and 12 AM. PND30 and PND60 females were killed in diestrus (assessed by vaginal smears).

Blood samples were collected in the morning, in EDTA-treated tubes, centrifuged at 3500 g for 20 min, and then the plasma was stored frozen at −80 °C. Samples were processed using standard procedures provided by manufacturers with the following kits: progesterone EIA-1561 (intra-assay variation (CV) is 5,4%, while the analytical sensitivity of this assay is 0.045 ng/mL), leptin ELI-4564 (intra-assay is 1,64% and inter-assay 3,96%, while the limit of sensitivity of this assay is 0.05 ng/mL (~3.13 pM) using a 10 μL sample size), total triiodothyronine (T3) EIA-4569 (intra-assay variation (CV) is 6.54% and inter-assay 5.23%, the analytical sensitivity of this assay is 0.1 ng/mL), ghrelin EZRGRA-90K (intra-assay variation is 1,60% and inter-assay 3.41%, while the limit of sensitivity of this assay is 8 pg/mL when using a 20 μL sample size)(DRG Instruments GmbH).

To minimize the stress for the animals, we measured testosterone levels in feces in young (PND30) and adult (PND60) male mice. Animals were isolated in a clean cage in the late morning. After 2 h 1.7 ± 0.3 mL of fecal pellet were collected, and animals returned to their home cage. Pellets were stored at −80 °C. Extraction of fecal testosterone was carried out on pulverized dried feces by using diethyl ether as previously reported [95]. Fecal testosterone level was determined using an enzyme immunoassay kit (K032; Arbor Assays, Ann Arbor, MI, USA) validated for multi-species dried fecal extracts.

### 4.4. Immunohistochemistry

#### 4.4.1. Fixation and Sampling

Mice were perfused at PND12, PND22, PND30, and PND60. Females at PND30 and PND60 were in diestrus (assessed by vaginal smear). Animals were deeply anesthetized with pentobarbital, monitored until the pedal reflex was abolished, and killed via intracardiac perfusion with saline solution (NaCl 0.9%) followed by fixative (4% paraformaldehyde, PAF, in 0.1 M phosphate buffer, pH 7.3). Dissected brains were stored in a freshly prepared PAF solution for 2 h at 4 °C, followed by washing in a 30% sucrose solution at 4 °C overnight. Finally, brains were frozen in liquid isopentane pre-cooled in dry ice at −35 °C and stored in a deep freezer at −80 °C until sectioning.

Three series of adjacent 40-μm-thick coronal sections were obtained with a cryostat (Leica CM 1900), collected in a cryoprotectant solution [96], and kept at −20 °C. We stained these three series respectively for kiss, POMC, and OX immunohistochemistry. To avoid between-assays variance due to systematic group differences, sections were processed into groups containing samples from each treatment and sex. Sections were washed overnight in PBS at pH 7.3 before immunohistochemical processing. The following day, after washing in PBS containing 0.2%Triton X-100 for 30 min the endogenous peroxidase activity was blocked with methanol/hydrogen peroxide solution (1:1) in PBS for 20 min at room temperature. Sections were then pre-incubated with normal goat serum (Vector Laboratories, Burlingame, CA, USA) for 30 min before the use of the specific antibodies. After the immunohistochemical reaction, the sections were collected on chrome alum pretreated slides, air-dried, washed in xylene, and cover slipped with Entellan mounting medium (Merck, Milano, Italy).

#### 4.4.2. Kisspeptin Immunohistochemistry

Kisspeptin immunostaining was performed according to our previous studies [24,56], by using an overnight incubation at 4 °C of floating sections with a rabbit polyclonal antibody (AC#566, Drs A. Caraty and I. Franceschini, Tours, France) at a dilution of 1:10,000 in PBS-Triton X-100 0.2%. Sections were then incubated, at room temperature, in biotinylated goat anti-rabbit IgG (Vector Laboratories) for 60 min at a dilution of 1:200. The antigen–antibody reaction was revealed with a biotin–avidin system (Vectastain ABC Kit Elite, Vector Laboratories, Burlingame, CA, USA) with an incubation of 60 min at room temperature. The peroxidase activity was visualized with 0.400 mg/mL of 3.30- diamino-benzidine (SIGMA-Aldrich, Milan, Italy) and 0.004% hydrogen peroxide in 0.05M Tris–HCl buffer pH 7.6. The specificity of the AC566 antibody for immunohistochemistry was previously reported [97].

#### 4.4.3. POMC Immunohistochemistry

POMC immunostaining was performed according to our previous studies [57,82], with an overnight incubation of floating sections at 4 °C with a rabbit polyclonal antibody against POMC (POMC precursor 27–52, H029-30, Phoenix Pharmaceuticals, Inc., Burlingame, CA, USA) diluted 1:5000 in PBS-Triton X-100 0.2%, pH 7.3–7.4. Sections were then incubated in biotinylated goat anti-rabbit IgG (Vector Laboratories, 1:250) for 60 min. The antigen-antibody reaction was revealed as described for kisspeptin immunohistochemistry. The POMC antibody specificity for immunohistochemistry was tested by the factory and in previous studies [57,82].

#### 4.4.4. Orexin Immunohistochemistry

Floating sections were incubated overnight in a rabbit anti-orexin A antibody (PC345, Calbiochem, Merck KGaA, Darmstadt, Germany) diluted 1:2000 in PBS at 4 °C. The sections were then incubated with biotinylated anti-rabbit IgG serum (Pierce, Vector, CA, USA, 1:200, 90 min). The antigen-antibody reaction was revealed as described for kisspeptin immunohistochemistry. The specificity of the orexin antibody for immunohistochemistry was tested by the factory and in previous studies [98].

In addition to the reported specificity, for each antibody we performed additional controls, omitting the primary antibody (negative control) or the secondary antibody. In these control sections, cells and fibers were completely unstained.

### 4.5. Quantitative Analysis

#### 4.5.1. Cell Counting and Fractional Area Evaluation

Based on the different nuclei and immunochemical markers, we evaluated the number of positive cells that were present in a nucleus or the extent of immunoreactivity (fractional area, FA), including cell bodies, dendrites, and fibers, within a nucleus. Digital images were acquired using a NIKON Eclipse 80i microscope (Nikon Italia SpA, Firenze, Italy) connected to a NIKON Digital Sight DS-Fi1 video camera. Images were then processed and analyzed with ImageJ (version 1.47v; Wayne Rasband, NIH, Bethesda, MD, USA). To have a better resolution, for each microscopic field, we took several pictures at different levels of focus collecting them in a stack of 4–7 pictures. These stacks were processed using the Z Project-Maximum intensity function of Image J, which created an output image, each of whose pixels contained the maximum value overall of the images in the stack, determining an optimal focus for all the structures contained in the stack.

Cell counting was performed only in those regions where cell bodies were clearly labeled with the specific antibody, in predetermined fields (region of interest, ROI). If cell bodies could be easily extracted from the background using the threshold function, the digitized images were processed and analyzed using the Analyze Particles automatic function of ImageJ (OX cells in LH nucleus); in the other cases we used the manual Image J Cell counter plugin (POMC cells in ARC nucleus).

The fractional area (FA) was evaluated, according to the general principles described by Mize et al. [99], by calculating the percentage of pixels covered by the immunopositive structures highlighted using the threshold function of Image J in a predetermined ROI. Due to differences in the immunostaining, the range of the threshold was individually adjusted for each section. By using the Analyze-Measure function of Image J the percentage of area covered by threshold within the ROI was automatically measured. The results were grouped to provide mean (±S.E.M.) values.

#### 4.5.2. Kisspeptin

For the immunostaining of the kiss system, three standardized sections were selected for each of the 3 analyzed nuclei, matching the Mouse Brain Atlas [59]: ARC (Bregma −1.58 mm, −1.70 mm, and −1.82 mm), PVN (Bregma −0.58 mm, −0.82 mm, and −0.94 mm), and RP3V (Bregma 0.26 mm, 0.02 mm, and −0.22 mm). Digital microphotographs were acquired with x40 objective (PVN) or x20 objective (ARC and RP3V) and were processed and analyzed with ImageJ (see above). Measurements were performed within predetermined ROIs. The PVN, in each selected section, was divided, as in our previous study [24], into fourteen squares (each of 31,100 µm^2^ at PND12; 37,050 µm^2^ at PND22; 40,150 µm^2^ at PND30 and PND60) to cover its full extension (see Figure 7a) and grouped in four regions: dorso-medial, dorso-lateral, ventro-medial, and ventro-lateral. The measure of total PVN was a mean of the FA measured in each of the four regions. The ROI for the ARC changed during development (at PND12 550,000 µm^2^, at PND22 600,000 µm^2^, and in adults at 890,000 µm^2^) to include the immunopositive region, and it was placed using the third ventricle as a reference to always have the same orientation. We easily identified and counted positive kiss neurons (characterized by the presence of a clearly labeled cell body) only within RP3V, while in the ARC and PVN, we quantified the FA covered by immunoreactive material.

#### 4.5.3. POMC

The number of POMC positive cells and the FA covered by immunoreactivity were analyzed in three selected standardized sections of comparable levels of ARC adjacent to the ones stained for the kiss, as well as the measurement of FA within PVN [59]. For ARC and PVN we used the same ROIs and analysis as for the kiss immunostained sections. For the dorsomedial hypothalamic nucleus (DMH), three standardized serial sections of comparable level were selected to analyze POMC-ir fibers (Bregma −1.46 mm; −1.82 mm; −1.94 mm); we acquired images using a 20× objective and used an ROI (1250 µm^2^) located within borders of the nucleus as evidenced by the immunoreactivity.

#### 4.5.4. Orexin

We measured the number of OX cells within four coronal sections through the region of the lateral hypothalamic area (LH; Bregma −1.06 mm; −1.34 mm; −1.58 mm; −2.06 mm) according to the Mouse Brain Atlas [59], which is where most OX-ir cells were found in the caudal hypothalamus [58]. The sections were acquired using a 10× objective. The OX-ir cells were counted within a ROI (1,580,000 µm^2^) that covered the entire extension of the nucleus. The results were grouped to provide mean (±S.E.M.) values. Furthermore, the FA was analyzed in three selected standardized sections of comparable levels of the PVN adjacent to the ones stained for POMC [59], and to measure OX-ir fibers, we used the same ROIs and analysis used for the kiss immunostained sections.

### 4.6. Statistical Analysis

Quantitative data were examined with SPSS statistic software (SPSS Inc, Chicago, IL, USA) via three-way ANOVA, where age, sex, and treatment were considered independent variables, or/and two-way ANOVA (considering sex and treatment or age and treatment as independent variables), and one-way ANOVA. When appropriated, we performed the Bonferroni or Tukey multivariate test to compare groups or Student’s *t*-test. The data are presented as mean ± SEM and the differences between groups are considered significant for values of *p* ≤ 0.05.

## 5. Conclusions

In conclusion, early postnatal exposure to GEN determines long-term sex-specific organizational effects on neural circuits controlling food intake, energy metabolism, and reproduction in CD1 mice, which are more pronounced in females. At the same time, other parameters related to reproduction are also altered (i.e., puberty and estrous cycle), as well as those related to metabolism (i.e., body weight, food consumption, and feed efficiency). Our hypothesis is therefore that these effects are strictly linked to alterations of neuroendocrine circuits controlling both reproduction and energy expenditure.

According to this view, GEN may be classified not only as an EDC with strong effects on reproduction but also as a metabolism-disrupting chemical (MDC). The danger to human health of synthetic contaminants in food, such as pesticides, is widely known and a subject of debate even among non-specialists. Much less known are the dangers associated with some molecules of natural origin, such as phytoestrogens, including GEN, that are present in many foods. Genistein, by binding estrogen receptors, can alter the functional processes that depend on them (for example, reproduction and energy metabolism) and the development of the neuroendocrine circuits that regulate these activities. The alteration of these nervous circuits could be at the root of some problems (constantly growing in our society) that are found in the human field, such as the predisposition to obesity in children fed with soy milk. Furthermore, the effects of GEN on development may be due to epigenetic modifications in the offspring. It is therefore important, for food safety and human health, to better investigate the effects of phytoestrogens on the central nervous system and the repercussions they can have in the organization of many nervous circuits regulated by hormones.

## Figures and Tables

**Table 1:** Plasma concentration of T3 and ghrelin. The concentration of T3 (expressed as ng/mL, mean ± SEM) and concentration of ghrelin in the plasma (expressed as ng/mL, mean ± SEM) at PND30 and PND60 for different groups of mice.

**Plasma Concentration of T3 (ng/mL)**				
	**M-CON**	**M-GEN**	**F-CON**	**F-GEN**
	(mean ± SEM)	(mean ± SEM)	(mean ± SEM)	(mean ± SEM)
**PND30**	5.3 ± 0.32	5.8 ± 0.28	5.6 ± 0.19	6.2 ± 0.34
**PND60**	6.4 ± 0.06	5.7 ± 0.11	7.5 ± 0.19	6.3 ± 0.09
**Plasma Concentration of ghrelin (ng/mL)**				
	**M-CON**	**M-GEN**	**F-CON**	**F-GEN**
	(mean ± SEM)	(mean ± SEM)	(mean ± SEM)	(mean ± SEM)
**PND30**	0.30 ± 0.04	0.40 ± 0.06	0.34 ± 0.07	0.7 ± 0.11
**PND60**	0.87 ± 0.18	4.23 ± 0.69	4.33 ± 0.73	2.76 ± 0.26

## Data Availability

All the data are available from the authors upon reasonable request. There are no restrictions on the data availability.

## Supplementary Materials

The following are available online at https://www.mdpi.com/article/10.3390/metabo11070449/s1, Table S1: Mammary gland length and terminal end bud (TEB) numbers, Table S2: Fecal testosterone concentration, Table S3: Kisspeptin system: quantitative data, Table S4: Body weight gain, Table S5: Daily food eaten, Table S6: Daily feed efficiency, Table S7: POMC system: quantitative data, Table S8: POMC and orexin system: developmental data, Table S9: Orexin system: quantitative data.
