# Supplementary material for: Early Postnatal Genistein Administration Affects Mice Metabolism and Reproduction in a Sexually Dimorphic Way
Source: Metabolites. 2021 Jul 10;11(7):449. doi: 10.3390/metabo11070449 (PMC8303179; doi:10.3390/metabo11070449)
Supplement: Supplementary file 1 [file metabolites-11-00449-s001.zip › TableS1-Gland length.pdf]

Gland length	F-CON	F-GEN
**PND22**	3.90±0.21	3.03±0.39
**PND30**	12.93±0.86	10.55±0.19
**PND60**	22.03±0.98	21.65±2.51
**TEB**		
**PND22**	10.50±4.5	13.00±1.80
**PND30**	11.67±1.33	13.50±0.65
**PND60**	NA	NA

Table S1: Mammary gland length and terminal end buds (TEB) numbers. Mammary gland length (mm) and TEB numbers in virgin female mice CD1 control (F-CON) or treated with Genistein (F-GEN) at PND22, PND30 and PND60 are reported in the corresponding columns (Mean±SEM).
