# Supplementary material for: Early Postnatal Genistein Administration Affects Mice Metabolism and Reproduction in a Sexually Dimorphic Way
Source: Metabolites. 2021 Jul 10;11(7):449. doi: 10.3390/metabo11070449 (PMC8303179; doi:10.3390/metabo11070449)
Supplement: Supplementary file 1 [file metabolites-11-00449-s001.zip › TableS2-Fecal Testosterone.pdf]

Fecal Testosterone (ng/gr)		
	**M-CON** (MEAN±SEM)	**M-GEN** (MEAN±SEM)
**PND30**	0.62±0.07	0.59±010
**PND60**	1.12±0.09	0.5±0.16

Table S2: Fecal testosterone. The concentration of fecal testosterone (expressed as ng/gr) at PND30 and PND60 for different groups of CD1 male mice is reported in the columns (Mean±SEM).
