# Supplementary material for: Early Postnatal Genistein Administration Affects Mice Metabolism and Reproduction in a Sexually Dimorphic Way
Source: Metabolites. 2021 Jul 10;11(7):449. doi: 10.3390/metabo11070449 (PMC8303179; doi:10.3390/metabo11070449)
Supplement: Supplementary file 1 [file metabolites-11-00449-s001.zip › TableS3-Kisspeptin.pdf]

Kisspeptin-ir (Fractional Area) in ARC

	M-CON (MEAN±SEM)	M-GEN (MEAN±SEM)	F-CON (MEAN±SEM)	F-GEN (MEAN±SEM)	ANOVA 1 WAY F	P
**PND12**	1.4±0.008	7.0±0.02	5.4±0.42	1.8±0.44	53.247	<0.001
**PND22**	1.9±0.179	2.4±0.32	12.0±0.89	8.7±0.42	64.367	<0.001
**PND30**	6.1±0.40	4.5±0.36	21.1±0.43	25.9±0.46	664.655	<0.001
**PND60**	9.2±1.33	8.9±0.31	25.2±1.53	12.9±0.51	58.391	<0.001

Kisspeptin positive cells in RP3V

	M-CON (MEAN±SEM)	M-GEN (MEAN±SEM)	F-CON (MEAN±SEM)	F-GEN (MEAN±SEM)	ANOVA 1 WAY F	p
**PND12**	1.8±0.12	2.3±0.20	2.6±0.19	4±0.29	19.146	<0.001
**PND22**	2±0.20	3.5±0.18	7.5±0.46	10.5±0.69	73.769	<0.001
**PND30**	1.67±0.10	2.8±0.23	12.9±1.57	20.2±1.29	79.593	<0.001
**PND60**	1.6±0.30	2.2±0.42	31.7±2.23	19.6±0.93	149.712	<0.001

Kisspeptin-ir fibers (FA) in PVN

	M-CON (MEAN±SEM)	M-GEN (MEAN±SEM)	F-CON (MEAN±SEM)	F-GEN (MEAN±SEM)	ANOVA 1 WAY F	p
**PND12**	1.4±0.17	1.6±0.27	0.9±0.29	1.1±0.21	3.468	0.059
**PND22**	1.3±0.15	1.2±0.19	3.2±0.18	3.8±0.28	40.045	<0.001
**PND30**	1.1±0.14	1.1±0.23	3.7±0.19	6.7±0.31	134.996	<0.001
**PND60**	0.9±0.13	1.2±0.20	6.6±0.38	3.5±0.49	73.344	<0.001

Kisspeptin-ir fibres (FA) within subdivisions of PVN

		M-CON (MEAN±SEM)	M-GEN (MEAN±SEM)	F-CON (MEAN±SEM)	F-GEN (MEAN±SEM)	ANOVA 1 WAY F	p
**PND12**	DL	1.2±0.27	1.1±0.20	0.4±0.12	0.3±0.09	6.31	0.011
	DM	1.4±0.13	1.6±0.20	0.9±0.26	1.2±0.16	1.78	0.214
	VL	1.1±0.21	1.1±0.12	0.7±0.16	0.5±0.10	3.731	0.049
	VM	1.6±0.10	2.1±0.15	1.3±0.47	1.5±0.09	1.411	0.296
**PND22**	DL	0.8±0.16	0.9±0.21	1.6±0.20	1.9±0.25	7.132	0.003
	DM	0.9±0.14	0.9±0.12	3.2±0.08	3.2±0.27	51.358	<0.001
	VL	1.0±0.13	1.1±0.26	1.7±0.25	2.1±0.26	4.737	0.014
	VM	2.1±0.26	1.7±0.21	4.7±0.33	6.0±0.50	33.322	<0.001
**PND30**	DL	0.5±0.13	0.5±0.18	1.4±0.14	2.6±0.17	39.361	<0.001
	DM	0.8±0.23	0.7±0.09	4.0±0.12	6.6±0.43	117.27	<0.001
	VL	0.4±0.07	0.7±0.23	1.8±0.33	2.5±0.71	5.385	0.014
	VM	1.7±0.12	1.9±0.42	5.7±0.46	10.9±0.36	142.079	<0.001
**PND60**	DL	0.3±0.09	0.4±0.08	1.2±0.32	0.5±0.11	9.864	0.007
	DM	0.6±0.07	1.1±0.24	5.2±0.71	4.1±0.43	92.729	<0.001
	VL	0.4±0.16	0.7±0.07	1.6±0.17	1.1±0.15	31.008	<0.001
	VM	1.9±0.34	2.0±0.37	12.5±0.76	6.0±1.03	135.474	<0.001

Table S3: Kisspeptin system: quantitative data. Quantitative data for kisspeptin-ir (FA) in ARC, for number of the kisspeptin cells in RP3V, and FA within PVN [divided in DorsoLateral (DL), DorsoMedial (DM), VentroLateral (VL), and VentroMedial (VM)], are reported in the corresponding columns (Mean±SEM) for different groups and different age of CD1 mice. The results of the one-way ANOVA (F and p values) are reported at the right.
