# Supplementary material for: Early Postnatal Genistein Administration Affects Mice Metabolism and Reproduction in a Sexually Dimorphic Way
Source: Metabolites. 2021 Jul 10;11(7):449. doi: 10.3390/metabo11070449 (PMC8303179; doi:10.3390/metabo11070449)
Supplement: Supplementary file 1 [file metabolites-11-00449-s001.zip › TableS4-Body weight gain.pdf]

						Body weight gain (gr)
	**M-CON** (MEAN±SEM)	**M-GEN** (MEAN±SEM)	**F-CON** (MEAN±SEM)	**F-GEN** (MEAN±SEM)	**ANOVA 1 WAY** F	p
**PND1**	1.83 ± 0.08	1.81 ± 0.05	1.71 ± 0.06	1.67 ± 0.07	0.695	0.566
**PND8**	4.88 3 ± 0.26	5.41 ± 0.16	4.28 ± 0.16	5.11 ± 0.16	3.084	0.051
**PND12**	6.63 ± 0.40	6.83 ± 0.30	6.13 ± 0.21	7.08 ± 0.17	0.974	0.424
**PND18**	9.10 ± 0.59	9.62 ± 0.50	8.27 ± 0.26	9.60 ± 0.33	1.015	0.407
**PND22**	13.18 ± 0.62	13.55 ± 0.80	12.11 ± 0.36	13.25 ± 0.43	0.579	0.635
**PND30**	26.08 ± 0.70	26.20 ± 0.69	20.32 ± 0.38	21.91 ± 0.10	15.614	0.001
**PND40**	32.05 ± 0.60	32.15 ± 0.55	23.67 ± 0.61	26.47 ± 0.24	33.589	0.001
**PND50**	34.32 ± 0.41	35.80 ± 0.71	25.13 ± 0.55	28.27 ± 0.35	45.681	0.001
**PND60**	36.30 ± 0.65	37.50 ± 0.58	25.7 ± 0.77	29.93 ± 0.65	34.64	0.001

Table S4: Body weight gain. Body weight gain (expressed in grams) measured during the development for different groups of CD1 mice, is reported in the columns (Mean±SEM). The results of the one-way ANOVA (F and p values) are reported at the right.
