# Supplementary material for: Early Postnatal Genistein Administration Affects Mice Metabolism and Reproduction in a Sexually Dimorphic Way
Source: Metabolites. 2021 Jul 10;11(7):449. doi: 10.3390/metabo11070449 (PMC8303179; doi:10.3390/metabo11070449)
Supplement: Supplementary file 1 [file metabolites-11-00449-s001.zip › TableS5-Daily food eaten.pdf]

Daily food eaten

	M-CON (mean±SEM)	M-GEN (mean±SEM)	F-CON (mean±SEM)	F-GEN (mean±SEM)	ANOVA 1 WAY F	*p*
**week1**	3.13±0.24	3.25±0.34	2.99±0.23	3.11±0.34	0.227	0.877
**week2**	5.03±0.26	4.97±0.09	4.16±0.16	4.06±0.30	5.523	0.006
**week3**	6.26±0.19	6.26±0.46	5.63±0.32	5.49±0.38	1.354	0.285
**week4**	5.80±0.20	5.50±0.05	4.75±0.23	4.72±0.08	11.832	0.001
**week5**	5.50±0.32	5.25±0.24	4.55±0.32	4.56±0.21	3.095	0.050

Table S5: Daily food eaten. Daily food eaten (expressed in grams) during five weeks after weaning for different groups of CD1 mice is reported in the corresponding columns (Mean±SEM). The results of the one-way ANOVA (F and p values) are reported at the right.
