# Supplementary material for: Early Postnatal Genistein Administration Affects Mice Metabolism and Reproduction in a Sexually Dimorphic Way
Source: Metabolites. 2021 Jul 10;11(7):449. doi: 10.3390/metabo11070449 (PMC8303179; doi:10.3390/metabo11070449)
Supplement: Supplementary file 1 [file metabolites-11-00449-s001.zip › TableS6-Daily feed efficiency.pdf]

Daily feed efficiency (Body weight/Kcal)

	M-CON (mean±SEM)	M-GEN (mean±SEM)	F-CON (mean±SEM)	F-GEN (mean±SEM)	ANOVA 1 WAY F	*p*
**week1**	1.23 ± 0.05	1.17 ± 0.06	1.19 ± 0.03	1.14 ± 0.04	0.245	0.864
**week2**	1.41 ± 0.05	1.44 ± 0.05	1.38 ± 0.02	1.39 ± 0.06	0.116	0.950
**week3**	1.40 ± 0.02	1.43 ± 0.03	1.24 ± 0.02	1.28 ± 0.04	4.064	0.021
**week4**	1.69 ± 0.03	1.78 ±0.03	1.50 ± 0.04	1.59 ± 0.03	5.959	0.004
**week5**	1.91 ± 0.02	2.08 ± 0.04	1.70 ± 0.03	1.78 ± 0.04	10.467	0.001

Table S6: Daily feed efficiency. Daily feed efficiency (expressed as body weight gain/Kcal) during five weeks after weaning for different groups of CD1 mice is reported in the corresponding columns (Mean±SEM). The results of the one-way ANOVA (F and p values) are reported at the right.
