# Supplementary material for: Early Postnatal Genistein Administration Affects Mice Metabolism and Reproduction in a Sexually Dimorphic Way
Source: Metabolites. 2021 Jul 10;11(7):449. doi: 10.3390/metabo11070449 (PMC8303179; doi:10.3390/metabo11070449)
Supplement: Supplementary file 1 [file metabolites-11-00449-s001.zip › TableS7-POMC System.pdf]

POMC System

	M-CON (MEAN±SEM)	M-GEN (MEAN±SEM)	F-CON (MEAN±SEM)	F-GEN (MEAN±SEM)	ANOVA 1 WAY F	p
**Cells in ARC**	33.33±2.88	23.9±2.96	30.2±2.78	43.2±3.94	7.708	0.002
**FA in ARC**	8.66±1.53	6.65±1.31	7.44±0.79	12.4±1.17	4.138	0.024
**FA in DMH**	4.69±1.02	5.81±0.84	5.91±1.36	7.01±0.21	3.519	0.038
**FA in PVN**	6.63±0.47	8.45±0.63	9.38±0.29	3.83±0.14	40.275	<0.001
DL	1.49±0.37	2.04±0.48	2.08±0.13	0.76±0.11	4.587	0.021
DM	6.10±0.63	7.85±0.82	10.15±0.41	3.26±0.39	25.715	<0.001
VL	2.22±0.53	3.42±0.71	4.28 ±0.37	1.38±0.38	6.170	0.008
VM	16.34±0.99	19.85±2.15	21.12±0.83	9.05±0.40	28.040	<0.001

Table S7: **POMC system: quantitative data.** Quantitative data for the number of POMC cells in ARC and for POMC-ir structures (FA) in ARC, DM, and PVN for different groups in adult CD1 mice are reported in the corresponding columns (Mean±SEM). The results of the one-way ANOVA (F and p values) are reported at the right.
