# Supplementary material for: Early Postnatal Genistein Administration Affects Mice Metabolism and Reproduction in a Sexually Dimorphic Way
Source: Metabolites. 2021 Jul 10;11(7):449. doi: 10.3390/metabo11070449 (PMC8303179; doi:10.3390/metabo11070449)
Supplement: Supplementary file 1 [file metabolites-11-00449-s001.zip › TableS8- POMC-Orexin develop.pdf]

A) Number of POMC positive cells in ARC during postnatal development

	M-CON (MEAN±SEM)	M-GEN (MEAN±SEM)	F-CON (MEAN±SEM)	F-GEN (MEAN±SEM)	ANOVA 1 WAY F	p
**PND12**	56.30±3.72	50.5±1.51	46.6±2.02	51.2±2.77	13.957	0.002
**PND22**	40.90±2.44	51.22±3.68	41.9±5.34	39.6±4.32	1.617	0.242
**PND30**	29.50±2.32	33.0±1.68	36.4±1.19	38.0±2.61	2.212	0.132
**PDN60**	33.33±2.88	23.9±2.96	30.2±2.78	43.2±3.94	7.708	0.002

B) Number of Orexin positive cells in LH during postnatal development

	M-CON (MEAN±SEM)	M-GEN (MEAN±SEM)	F-CON (MEAN±SEM)	F-GEN (MEAN±SEM)	ANOVA 1 WAY F	p
**PND12**	190.30±8.27	160.6±5.48	177.7±10.89	182.8±10.57	2.346	0.111
**PND22**	225.7±12.06	191.9±6.57	216.1±7.24	179.1±9.85	5.472	0.008
**PND30**	316.9±11.72	210.3±16.94	288.8±20.62	343.9±27.31	8.346	0.001
**PND60**	313.7±18.21	253.4±13.52	189.9±19.29	260.70±10.90	8.904	0.001

Table S9: Postnatal changes in the average number of POMC and Orexin positive cells in ARC (A) and LH (B).
