# Supplementary material for: Early Postnatal Genistein Administration Affects Mice Metabolism and Reproduction in a Sexually Dimorphic Way
Source: Metabolites. 2021 Jul 10;11(7):449. doi: 10.3390/metabo11070449 (PMC8303179; doi:10.3390/metabo11070449)
Supplement: Supplementary file 1 [file metabolites-11-00449-s001.zip › TableS9-Orexin positive cells in four levels of LH.pdf]

A) Orexin positive cells in whole LH and in the selected four levels

	M-CON (MEAN±SEM)	M-GEN (MEAN±SEM)	F-CON (MEAN±SEM)	F-GEN (MEAN±SEM)	ANOVA 1 WAY F	p
**Average number**	313.7±18.21	253.4±14.70	189.9±19.29	260.70±10.90	8.904	0.001
Level 1	293.2±15.59	218.17±13.52	146.4±16.59	176.17±13.73	14.907	0.001
Level 2	402.8±23.14	339.33±16.69	189.8±21.20	278.17±14.01	19.866	0.001
Level 3	322.4±27.12	282.00±22.65	232.0±28.60	368.30±29.40	3.98	0.024
Level 4	236.6±14.93	174.17±27.11	191.4±21.67	220.17±19.42	1.652	0.213

B) Orexin-ir (Fractional Area) in PVN

	M-CON (MEAN±SEM)	M-GEN (MEAN±SEM)	F-CON (MEAN±SEM)	F-GEN (MEAN±SEM)	ANOVA 1 WAY F	p
**FA in PVN**	4.64±0.35	3.29±0.30	3.84±0.46	5.18±0.18	6.390	0.008
DL	4.35±0.54	3.66±0.27	3.60±0.38	4.32±0.56	0.801	0.517
DM	5.99±0.80	4.01±0.67	3.87±0.57	5.49±0.48	2.705	0.092
VL	4.06±0.22	2.26±0.71	3.78 ±0.62	4.71±0.82	2.649	0.096
VM	4.02±0.19	2.61±0.13	4.30±0.50	5.65±0.68	8.127	0.003

Table S8: Orexin system in adult mice (PND60):

A) The first row shows the average number of orexin-positive cells in the LH. The following rows show the mean amount of positive cells in the selected four levels of LH.

B) Quantitative data for Orexin-ir fibers (FA) within PVN [divided in DorsoLateral (DL), DorsoMedial (DM), VentroLateral (VL), and VentroMedial (VM)] are reported in the corresponding columns (Mean±SEM) for different groups of adult CD1 mice. The results of the one-way ANOVA (F and p values) are reported at the right.
